# PDR with a Foot-Mounted IMU and Ramp Detection

**DOI:** 10.3390/s111009393

**Published:** 2011-09-29

**Authors:** Antonio R. Jiménez, Fernando Seco, Francisco Zampella, José C. Prieto, Jorge Guevara

**Affiliations:** Centre for Automation and Robotics (CAR), Consejo Superior de Investigaciones Cientificas (CSIC)-UPM, Ctra. Campo Real km 0.2, La Poveda, Arganda del Rey, Madrid, 28500, Spain

**Keywords:** indoor localization, pedestrian dead-reckoning, inertial measureent unit (IMU), inertial navigation, drift elimination, ramp detection

## Abstract

The localization of persons in indoor environments is nowadays an open problem. There are partial solutions based on the deployment of a network of sensors (Local Positioning Systems or LPS). Other solutions only require the installation of an inertial sensor on the person’s body (Pedestrian Dead-Reckoning or PDR). PDR solutions integrate the signals coming from an Inertial Measurement Unit (IMU), which usually contains 3 accelerometers and 3 gyroscopes. The main problem of PDR is the accumulation of positioning errors due to the drift caused by the noise in the sensors. This paper presents a PDR solution that incorporates a drift correction method based on detecting the access ramps usually found in buildings. The ramp correction method is implemented over a PDR framework that uses an Inertial Navigation algorithm (INS) and an IMU attached to the person’s foot. Unlike other approaches that use external sensors to correct the drift error, we only use one IMU on the foot. To detect a ramp, the slope of the terrain on which the user is walking, and the change in height sensed when moving forward, are estimated from the IMU. After detection, the ramp is checked for association with one of the existing in a database. For each associated ramp, a position correction is fed into the Kalman Filter in order to refine the INS-PDR solution. Drift-free localization is achieved with positioning errors below 2 meters for 1,000-meter-long routes in a building with a few ramps.

## 1. Introduction

The location of people in outdoor environments is in general possible with a GPS receiver. However, indoors, the precise location of people is still a problem that needs more research to be totally solved. There are many solutions based on the deployment of a network of sensors in a building, which are used to estimate the person’s location by triangulation or multilateration approaches. These solutions are known as Local Positioning Systems (LPS) [1,2]. There are other solutions that, in principle, only require the use of an inertial sensor mounted on the person’s body, called Personal Navigation Devices (also known as PDR, from *Pedestrian Dead Reckoning*). Some PDR solutions basically integrate the signals coming from an Inertial Measurement Unit (IMU), which generally includes three accelerometers and three gyroscopes, and add special constraints for sensor error estimation [3]. Other PDR approaches use the inertial sensors for gait analysis, which can be used for modeling the walk parameters such as stride length and the direction of movement [4]. The main problem of PDR is the accumulation of positioning errors due to the drift caused by the imperfections and the noise in the IMU sensors [5].

Different approaches can be used to eliminate the positioning drift in PDR solutions [5]: (1) integration with external sensor systems, such as ultrasonic or radio-based LPS [6] (it implies additional costs for the installation of the infrastructure); (2) the use of other sensors onboard the person, such as, barometers, compasses, cameras, *etc*. [7] (they reduce drift but do not totally eliminate it); and (3) methods that apply movement constraints, such as straight-line path assumptions [8], fitting the position to accessible areas in the environment (map-matching) [9], or *action recognition* methods that classify the type of activity of the person [10].

**Figure 1.**
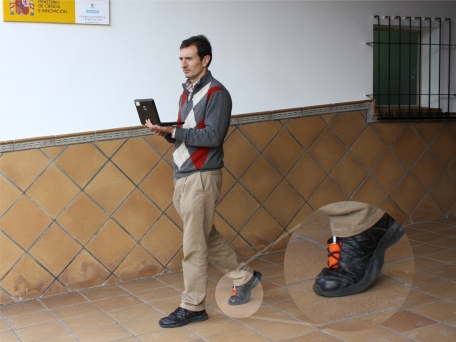
Person walking on one of the access ramps of CAR-CSIC building. For position estimation and ramp detection, an IMU is attached to the right foot of the person using the shoe laces (orange color box).

The main idea behind *action recognition* is to be able to detect what a person is doing at a particular instant. For example detecting whether a person is walking, sitting on a chair, lying on a bed, going upstairs, or standing in a lift. This information can be used for the assessment of the physical activity performed by a person (e.g., in health monitoring applications, in dangerous fire-fighting missions, *etc.*). It can also be used to select a movement model in a PDR implementation (e.g., walking at a continuous pace), and even more importantly, action recognition can be used to get clues about where a person could be located, allowing to make position corrections to eliminate drift. This latter approach is the one that we exploit in this work. In particular, we propose to detect with an IMU if the person is on a *ramp*, and if so, correct the PDR estimated position with the position of that ramp (see Figure 1 for a person walking on a ramp in our building).

There are some previous works in *action recognition* to detect many different states: walking, running, standing, sitting, falling, lying, going upstairs, going downstairs, as proposed by Korbinian and Vera [10,11]. In these works they use the signals of an accelerometer placed at different locations in the body (waist, chest, leg, arm) to extract some discriminant features that are used to classify the different *actions* in real-time (with a 90% success rate). Altum [12] proposed to classify 19 different actions, placing a total of 5 IMU on the body. Apart from the actions already mentioned they include: standing in a lift, on a conveyor belt, on a sports treadmill, riding a bike, jumping, rowing, *etc*. None of these works really apply action recognition to correct the drift in PDR, nor propose a method for *ramp* detection.

The works in the literature that are closer to our contribution, since they propose a position correction based on the recognition of actions that only can occur at particular locations, are the ones by Gusenbauer [13] and Kourogi [14]. In [13] the detection of elevators and escalators using the readings from an IMU is proposed. The method named “Activity based map-matching” applies positioning corrections in a direct Kalman filter whenever the person is detected on an escalator or in an elevator. The position correction is made with the position of the closest stair or lift. In [14] the detection of actions such as still, lateral walk or going upstairs/downstairs is used to improve the PDR algorithm. Besides, the improved position estimate is used to better detect the actions. No implementation details are given in any of these references.

The only reference that considers a ramp detection to correct positions is the one proposed by Wagner [15]. In this work, a map-matching is performed for cars in garages, and it is mentioned that the ramp detection is just used to initialize the car position when it enters into a garage. In this case, the inertial sensor is onboard the car, the slopes to detect are significant, and the ramps are tens of meters long. However, again, there are no implementation details neither about the ramp detection method, nor about how the position correction is actually performed. In fact, the paper focuses on the map-matching algorithms using a graph to represent the pathways for a car in a parking area. A straightforward method to detect ramps could consist in sensing the gravity component on the tri-axial accelerometer, that is, using the IMU as an inclinometer (valid if the sensor is quasi static, *i.e.*, the acceleration caused by motion is low compared to gravity).

In our paper, we present a method to correct the estimated position of a person based on the detection of ramps using only an inertial sensor. Employing the algorithmic framework for inertial-based PDR navigation proposed by Foxlin [16] and Jiménez [17], we add a ramp detection method that triggers position corrections whenever a person is detected on one of the ramps of the building. So, this work assumes that there exist access ramps in the building to connect areas at different height levels. Our proposal provides drift corrections in PDR without having to employ additional external absolute positioning sensors. The method is based on some algorithms that detect ramps by measuring the ramp slope and the change in height between consecutive steps. Then, ramps are evaluated for association with one with similar features in a pre-stored ramp database. We believe that we are the first authors to propose the detection of ramps using an IMU attached to the foot of a person [18].

Section 2 presents the PDR method including the ramp detection and the association algorithms. Section 3 shows the evaluation results for several indoor navigation tests. Finally, in last section, we give the main conclusions drawn from this work.

## 2. The IMU-based PDR Method with Ramp Detection

### 2.1. The Inertial Framework for PDR

The PDR algorithm that we use to integrate the IMU readings is the one recently proposed by Jiménez *et al*. [17], named IEZ+. As Foxlin [16] proposed, the use of a complementary Extended Kalman Filter (EKF) and a foot-mounted IMU has many benefits in PDR. For example, step detection is more reliable, and it is possible to apply Zero Velocity Update corrections (ZUPT) every time the foot is motion-less on the floor while walking (stance phase). We also perform update corrections to the EKF using Zero Angular Rate Updates (ZARU) when we detect that the foot is completely stationary (still condition) [17]. Whenever the IMU contains magnetometers, as in our case, it is possible to obtain acceptable heading corrections if the local Earth magnetic field is not significantly perturbed. It is recommended to calibrate the magnetometers in the IMU just after its installation on the foot, in order to compensate for soft and hard iron effects caused by persistent close-by metallic objects (e.g., by using the circular curve-fitting method [19]). The used IEZ+ PDR method provides position estimates with a limited drift, even using a typical low-performance MEMS sensor (MTi from Xsens).

**Figure 2.**
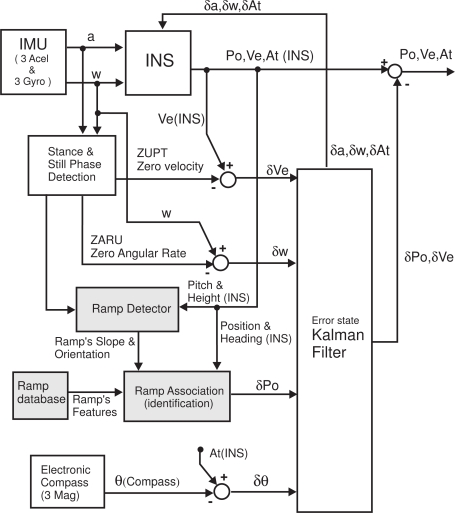
Block diagram of the PDR method with corrections based on ramps’ position.

The Extended Kalman Filter (EKF) works with a 15-element error state vector: **X** = [*δ*At, *δω^b^*, *δ*Po, *δ*Ve, *δ*a*^b^*]. This vector contains the estimated bias of accelerometers and gyroscopes (*δ*a*^b^* y *δω^b^*, respectively), as well as the 3D errors in attitude (*δ*At), position (*δ*Po), and velocity (*δ*Ve). Figure 2 represents a block diagram of the IEZ+ method, with some additional blocks (light-gray color) for ramp detection.

The Kalman filter provides error estimations at the update rate of the IMU (100 Hz). While the foot is stationary, totally or at stance intervals, there are several measurements available (ZUPT, ZARU and Compass) that are used to update the estimations in the EKF; while the foot is on the swing phase, instead of filtering, the EKF predicts errors. The Inertial Navigation System (INS) block always corrects its navigation output with the errors that are filtered or predicted by the EKF.

The IEZ+ PDR method, using only inertial information from one IMU, has proved to be very reliable with an accumulation of positioning errors of about 1%–2% of the Total Traveled Distance (TTD) [16,17]. However, as any integrating or dead-reckoning method, even with this small error, the accumulated error can be very significant for long distance routes (e.g., 10 meter error for 1,000-meter-long paths). In this work we propose a ramp correction method in order to achieve a drift-free PDR solution (subject to the existence of ramps in the building), as it is detailed in next subsection.

### 2.2. Position Correction with Ramps

We correct the drift by detecting when the user is located on a ramp, then identifying the ramp and consequently its position, and finally generating a position correction in the EKF used in our PDR implementation. As it can be seen in the light-gray blocks in Figure 2, a database contains the location of the ramps, their dimensions, orientations and slopes. The blocks termed “Ramp Detector” and “Ramp Association” are described in detail next.

#### 2.2.1. Ramp Detection

By definition a ramp can be distinguished from a leveled terrain by its slope. Also, when walking along a ramp, there should be a change in height (an ascent or descent). However, it is not so straightforward to detect a ramp while a person is walking by simply using a foot-mounted IMU. Some of the challenges are to detect low-slope ramps with noisy IMU signals, or to distinguish between inclinations coming from real ramps, and those due to irregular stances over uneven terrain.

With a suitable signal processing, two main parameters could be useful for ramp detection: (1) the pitch angle (*ψ*) of the IMU at each step; and (2) the difference in the vertical position (height) between two consecutive steps (*δz* or rise). At a ramp a positive rise has associated a negative pitch angle, and viceversa. So, we propose to detect a ramp when the product of both values (pitch and rise), which should be a negative number, is below a given threshold:

(1) $$\text{RampDetected}\;(k)=\{\begin{array}{ll} 1 & \psi (k)\cdot \delta z(k)<\text{Th}\\ 0 & \text{Otherwise} \end{array}$$

where k is the index of the step detected.

The pitch angle used in Equation (1) (*ψ*) is obtained, after some additional processing, from the pitch angle (*ψ̃*) at the output of the INS mechanization. This angle (*ψ̃*) measures the terrain inclination but also a pitch that only depends on how the IMU was attached to the foot (a factor which should be removed). The calculations to obtain the pitch of interest are: (1) filter the pitch angle with a FIR filter over the last three step samples (which adds a group delay of one step), *ψ_f_* (*k*) = 0.33*ψ̃*(*k*) + 0.33*ψ̃*(*k* − 1) + 0.33*ψ̃*(*k* − 2); (2) compute the IMU’s installation pitch as the average of the IMU pitch at each step with a first-order low-pass IIR filter, *ψ̄*(*k*) = 0.99*ψ̄*(*k* − 1) + 0.005*ψ_f_* (*k*) + 0.005*ψ_f_* (*k* − 1) (we assume that the path is in its majority over a leveled surface); (3) Finally, we obtain the inclination angle of the terrain (independent of IMU installation) as:

(2) $$\psi (k)=\psi_{f}\;(k)-\bar{\psi }(k)$$

The rise or change-in-height value between two consecutive steps, *δz*(*k*), is computed similarly as the pitch angle (in terms of filtering). In this case, the real height change has to be distinguished from the rise caused by the vertical drift at the INS output. The detrended change in height between two consecutive steps is:

(3) $$\delta z(k)={\delta z}_{f}\;(k)-\bar{\delta }z(k)$$

where *δz_f_* (*k*) is the rise between two steps computed as: *δz_f_* (*k*) = *z_f_* (*k*) − *z_f_* (*k* − 1), and *δ̄z*(*k*) is the estimated drift in height.

The threshold (Th in Equation (1)) is chosen as 9 degrees·cm. This threshold was selected to detect ramps with inclinations larger than 2 degrees; for this threshold calculation we had taken into account that a typical stride length (distance between two consecutive right-foot stances) is about 1.3 m ([tan(2) · 130] · 2 = 9 degrees·cm). If targeting the detection of ramps with inclinations lower than 2 degrees, then false detections could become a problem. It is important to remark that this design (detections of ramps with inclinations larger than 2 degrees) does not necessarily mean that foot stances over leveled but irregular terrain (e.g., a paved path) causes many false detections; this is because if no significant changes in height are simultaneously detected, then the ramp would not be detected. In principle, while walking on an leveled but irregular terrain (with a maximum of 1.5 cm rise between two consecutive steps), the method supports foot stances with inclinations up to 6 degrees without detecting them as a ramps. The performance of the ramp detection method under this challenging irregular terrain case is analyzed in Section 3.4.

#### 2.2.2. Ramp Association

**Figure 3.**
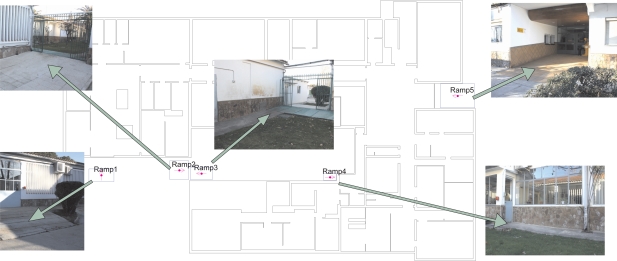
Ramps to access the main building of CAR-CSIC center.

Once a ramp is detected with the above-mentioned method, it is necessary to identify the particular ramp where the person is located. To do that, the estimated position of the person (from the INS output) is compared with the ramp positions contained in the database, which correspond to their geometrical centers. In order to avoid ambiguities, the association is done with the closest ramp in the database with an orientation or heading similar to the direction of movement of the person (within ±30°). The ramp’s orientation (marked with a magenta arrow in Figure 3) defines the direction of ascension along the ramp, and the pedestrian’s direction of ascension is known from the parameter (*δz*) that indicates whether the person is going up or down.

In order to avoid false associations caused by false ramp detections, no associations are performed if the distance between the detected ramp and the one in the database is larger than a threshold (5 m in our implementation). Taking into account that the IEZ+ has a drift of about 1%–2% of the total traveled distance [17], a threshold selection of 5 m indicates that, in principle, a new position correction by ramp detection is needed when the path-length reaches 250 m. In practice, it would be necessary to update the position approximately every 150 m, since the position correction, in the EKF filter, is not complete after a single ramp detection (the certainty about the correction is limited).

#### 2.2.3. Position Correction

After the ramp association, the pre-stored position of the center of the ramp in the database, Po_ramp_, should be similar to the estimated position at the INS output (Po). However, in general, due to the typical PDR drift both positions can differ significantly from each other. This difference gives an indication of to the positioning error (*δ*Po), which will be used to feed the EKF (Figure 2):

(4) $$\delta \text{Po}(k)=\text{Po}(k-1)-{\text{Po}}_{\text{ramp}}(k)$$

where the correction is done with the position of the last detected step, because the ramp detector added a one-step delay at the FIR filter.

The maximum frequency at which this error correction is fed into the EKF filter is once every step detected over a ramp. If the ramp is long enough, it is possible to get several steps over the same ramp, and therefore there could be several updates (at the step frequency ≃1 Hz). If the user stops on a ramp, there is only one ramp update at the beginning of the stance/still condition, *i.e.*, ramp updates depend on the number of steps on the ramp, not on the time spent on them.

The certainty that we have on the correction error measurement (*δ*Po), depends on several factors such as: the ramp size, the similitude between the heading of movement and the orientation of the ramp, or even their slope similitudes. In this work, in a first approach, we use a standard deviation that is proportional to the size of the ramp ( $\sigma_{\delta \text{Po}}=\sqrt{\text{Length}\cdot \text{Width}}/4$). This means that the smaller ramps are the ones with higher certainty. On the contrary, wider ramps do not provide a strong correction because the user could be located in different subareas along the ramp. This certainty could be increased for larger ramps if it were designed a method to estimate the position within the ramp based, for example, on the total ascension over a particular ramp, or by tracking the transients between the leveled and the sloped floor.

## 3. Experimental Evaluation

### 3.1. Test Conditions

In this section we firstly describe the features of the ramps in our building, then the used Inertial Measurement Unit (IMU) is presented, and finally, we give a general description of the navigation tests.

#### 3.1.1. The Ramps in the Building

Tests were performed in the main building of our working place, the Center for Automation and Robotics (CAR-CSIC), which has several access ramps at the transitions from indoors to outdoors. In Figure 3 there is a floor plan of the building with the location of the ramps. The features of these ramps are detailed in Table 1.

#### 3.1.2. The Sensor

The inertial sensor used is the MTi model from the company XSens Technologies B.V. (www.xsens.com). It has a size of 58 × 58 × 22 mm, and it weights 50 grams. It is configured to output data from each triad of accelerometers, gyroscopes and magnetometers at 100 Hz. This IMU is mounted on the user’s foot (required to use the IEZ+ algorithm), attaching it to the right shoe with its own shoe laces.

#### 3.1.3. Navigation Tests

Several navigation tests were performed by one person (with a height of 1.8 m) walking at a normal speed (1.2 m/s on average) inside our main building and with frequent transitions to outdoor areas. We used all the five main access ramps in the building (shown in Figure 3). The performance results given in next subsections are based on these data-collection experiments that accounts for a total of 2120 meter-long paths and 1476 detected steps, 120 of which on ramps.

### 3.2. About the Metric to Detect Ramps

**Figure 4.**
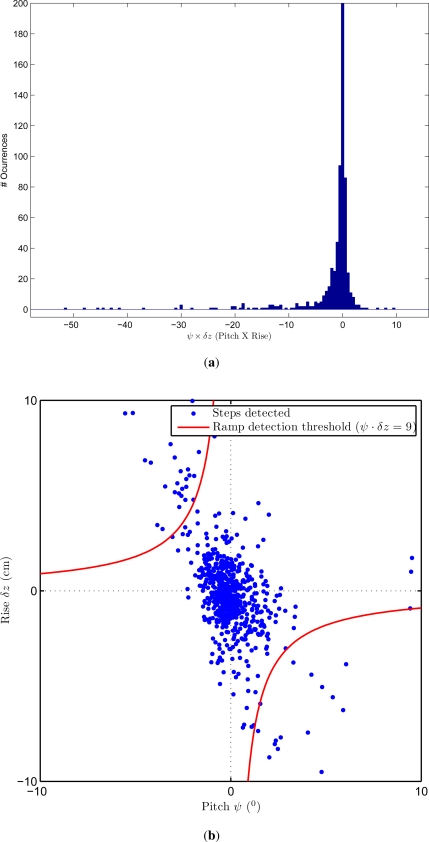
The metric to detect ramps (*ψ* · Δ*z*, Pitch multiplied by Rise): (**a**) Histogram of the metric for a total of 696 steps, and (**b**) Distribution of all detected steps on a Pitch versus Rise plot (the threshold 9 degrees·cm, used for ramp detection, is overlayed).

Above, we presented a simple metric *ψ*·Δ*z* to decide whether a step is on a ramp or not. We assumed that a threshold value of about 9 degrees·cm should be adequate to detect ramps with an inclination over 2 degrees (typical SL of 1.3 m). Also, we highlighted that this metric should have only negative values for ramps. However, for the case of flat terrain, this metric should be a random value around zero (positive or negative). In the histogram of *ψ* · Δ*z* presented in Figure 4(a) this fact can be clearly visualized, *i.e.*, most steps have a *ψ* · Δ*z* value around zero with a standard deviation of 1.6 degrees·cm. A long tail appears along the negative values of the metric (from −5 to −55) that corresponds mainly to the detected ramps.

The fact that the *ψ* · Δ*z* metric should have only negative values for ramps means that there is a correlation between *ψ* and Δ*z*, *i.e.*, positive values of Pitch (*ψ*) come with negative values of Rise (Δ*z*), and vice versa. This correlation for ramps is shown in Figure 4(b), as a point spreading from quadrant II to IV (*i.e.*, from “North-West” to “South-East” in the plot). On the other hand there is also a larger group of uncorrelated steps centered around (0,0) that corresponds to the steps on a flat surface. It can be seen that the heuristically selected threshold (Th = 9 degrees·cm) is reasonable to separate the uncorrelated flat steps from the correlated steps on ramps.

It is important to remark that if our navigation tests would have included stairs, then the Rise value (Δ*z*) would be high (more than 15 cm), causing the metric *ψ* · Δ*z* to frequently overpass the ramp threshold (Th). Since we do not want to detect stairs as if they were ramps, then the basic condition for ramp detection (Equation (1)) should be augmented to reject those steps with a significant Rise and almost null Pitch.

### 3.3. Ramp Detection Performance

The evaluation tests indicate that it is possible to detect each of the 5 ramps in the database, including those with low slopes (ramp numbered 2, 3 and 4). We analyzed the percentage of correct ramp detections for each of the 5 tested ramps in our building, which is of 100% for ramps 1, 2, 3 and 5, and 76% for ramp number 4 (the ramp with the lowest slope). Consequently, the ramp number 4 was mis-detected in 24% of the cases in our tests. The percentage of false alarms (steps not on ramps that are detected as ramps) was only 0.15% with respect to the total detected steps. False detection of ramps occurs in some cases (e.g., due to irregular stances over the border of a carpet), but in almost all cases, these false ramps are rejected during the association, and therefore not used to update the Kalman filter.

The detection data above-described corresponding to our selected threshold (Th = 9 degrees·cm) is presented in Table 2 (2nd row), together with detection probabilities for other thresholds (5, 15 and 30 degrees·cm). It is clear, as expected, that a lower threshold servers to detect 100% of ramps but with the cost of generating about a 3% of false alarms (not desirable). An increase of the threshold causes the elimination of all false alarms, but the amount of mis-detections increases, *i.e.*, less ramps are detected (85% ramps detected for Th = 15, and 68% for Th = 30). So a value of 9 for Th seems to be a good compromise for ramp detection.

**Figure 5.**
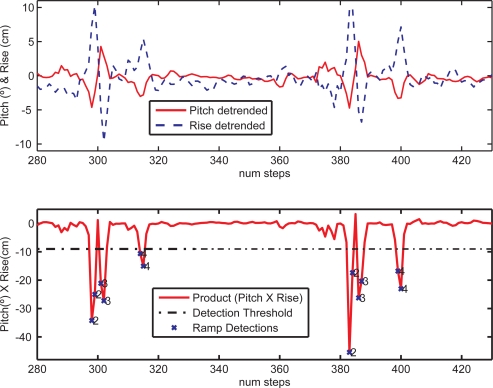
Ramp detections and associations, for 2 repetitions of a test visiting ramps 2, 3 and 4. Top graph: Estimated Pitch and Rise. Bottom graph: Ramp detections (crosses) and the identification of ramps after the association (marked with the number of the associated ramp).

As a significative example of the capacity to detect and associate ramps, the graph of Figure 5 is very illustrative. In this particular test, ramps 2, 3 and 4 are visited along a cyclic route repeated twice. The individual values of the estimated slope (*ψ*) and rise (*δz*) are also shown, as well as the product of both values (metric to be thresholded). As can be expected, several ramp detections occur on the same ramp, as many as right-foot stances on that particular ramp. The numbers in the plot near the detected ramps (crosses) are the results of the association. Association works perfectly well even if significant false alarms appear (assuming that these false alarms do not occur at a position close and similar to a ramp in the database). No false ramp association occurred in any of our tests.

### 3.4. Ramp Detection Walking over Irregular Terrain

**Figure 6.**
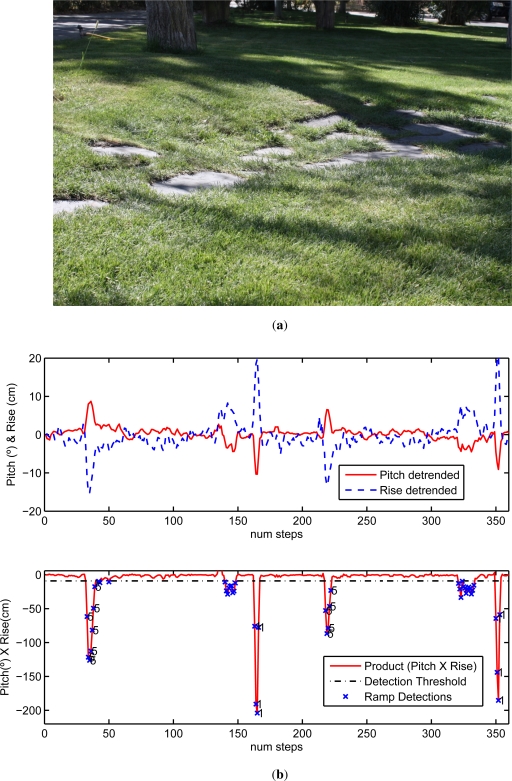
Ramp detection over irregular terrain: (**a**) Photograph of the uneven trail made of stones placed over the grass; (**b**) Ramp detections and associations, for 2 repetitions of a test visiting ramps 1 and 5, as well as the uneven trail; top graph: Estimated Pitch and Rise; bottom graph: Ramp detections (crosses) and the identification of ramps after the association (marked with the number of the associated ramp).

Results presented in last subsection correspond to walking tests over typical indoor floors (flat and leveled) and some access ramps with quite continuous surfaces. When presenting the methods to detect ramps (Section 2.2) we stated that it would be challenging to distinguish between inclinations coming from real ramps and those due to irregular stances over uneven terrain. To study this, some of our tests included walking over grass with a path made of stone tiles sparsely placed over the ground; this trail (see Figure 6(a)) has diverse inclinations and small height changes that represent a real challenge for our ramp detection method.

An example of the ramp detection performance is shown in Figure 6(b); here apart from ramps 1 and 5 that are detected easily (ramps 1 and 5 have a large inclination), there are several detected false alarms (in step numbers: 140–150 and 330–340) that corresponds to walking along the uneven terrain of Figure 6(a). These false alarms, although undesirable, do not generate false associations (as can be seen in the figure); in fact, these false ramps are rejected or ignored during the association phase, since no similar ramps in the database are close to the uneven trail. It could be possible to have irregular terrain close to real ramps, in that case, the best option would be to modify the threshold to a larger value (e.g., Th = 30) to eliminate most false alarms, although in that case, some low slope ramps would be undetectable e.g., ramp number 4 (see last row of Table 2).

### 3.5. Drift Corrections in Position

**Figure 7.**
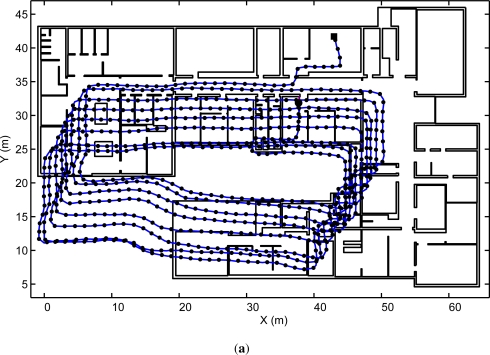

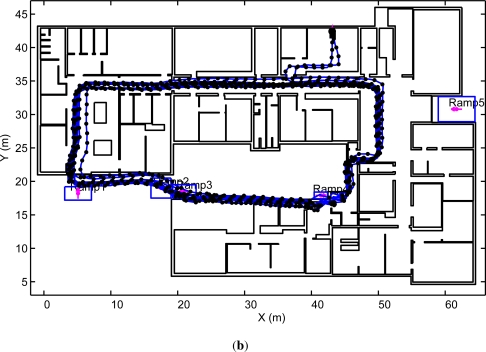
Closed route repeated 8 times, for a trajectory with a total length of 1 km. The trajectory is along the main corridors in the CAR-CSIC building, going temporarily to an exterior yard to finally enter again in the building. (**a**) PDR estimation with IEZ+ algorithm (a significant drift is observed). (**b**) Ramp-assisted PDR estimation (the drift is eliminated).

In our tests several closed routes were repeated until a significant positioning drift was accumulated using only the IEZ + PDR method without ramp aiding (*i.e.*, a pure dead-reckoning solution). When the algorithms proposed in this paper were activated to use the ramp corrections, we obtain almost a perfect elimination of the accumulative positioning errors (0.15% of the total traveled distance; TTD). Figure 7 shows one route that is 1,000 meters long where ramps 2, 3 and 4 are visited eight times each (some ramp detection details of this particular test are available in Figure 5 for 2 of the 8 cycles). Figure 7 shows that the position correction is effective with a frequent ramp visit, however the final positioning error should depends on the walked distance since the last detected ramp.

**Figure 8.**
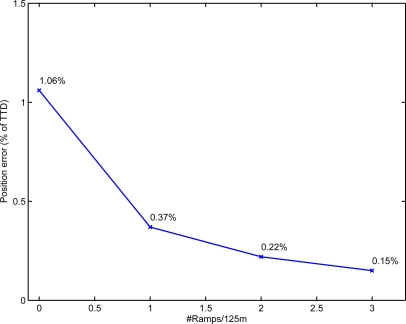
Influence of the Ramp density on the final position error.

We analyzed the dependance of the final positioning error (in terms of Total Traveled Distance-TTD) with respect to the density of ramps used in the building. In Figure 8 the result of this study is summarized. As expected the addition of more ramps in the building helps to reduce the final positioning error. The best results are obtained with all the ramps in the test (0.15%) that corresponds to a ramp density of 3 ramps every 125 m of path on average. With only one ramp every 125 m, the method is still effective (0.37% of TTD), specially if compared to the case when no ramps are used (1.06% of TTD).

### 3.6. Discussion about Tests and Results

This paper restricted the evaluation tests to one particular person carrying the IMU on the foot. We know that the particular height of a person (leg’s length) and his walking speed, can cause different Stride Lengths (SL). There is a clear influence of a particular Stride Lenght (SL) on the computed Rise (Δ*z*) parameter when walking on a ramp (computed as the difference in height of two consecutive steps). However the metric to detect a ramp takes into account two terms (Rise and Pitch, in fact their product: *ψ* · Δ*z*), and fortunately the pitch term is not influenced by the type of person, so in practice the different SL of particular persons is expected to have a very limited influence on the detection of ramps; of course, assuming that the person walks at a normal pace (0.5–1.5 m/s).

The influence of the different SL of a person would affect, as explained above, to the Rise parameter. Shorter SL would cause lower Rise, and a larger SL would cause a larger Rise. Pitch however would be identical with independence of the person. As can be deduced from Figure 4(b), a change in the Rise parameter would cause a compression or dilatation on the vertical axis of this figure. This effect is somehow similar to a rotation of the points spread along the axis from quadrant II to IV, CCW if SL is lower (less Rise) or CW if SL grows. As can be seen in Figure 4(b), the area covered by the threshold is wide compared with the correlated candidate ramp steps, so this SL effect is not expected to be important. This fact will be studied in future works and in case that we need a Rise term more independent from SL, we believe that a normalization with an estimated SL value can be used (Δ*z*/SL).

## 4. Conclusions

We have presented an algorithm that detects *access ramps* in buildings and, with that information, corrects the positioning drifts in PDR solutions. It only requires the use of one IMU attached to the foot of a person. We know that the proposed method is a “partial” solution to the problem of drift elimination in PDR, because it assumes that a building must have some ramps in frequently-visited places. However, our proposal should be a very useful complement in a final PDR solution that integrates multiple methods to compensate drift (e.g., integrated with map-matching, Radio-based or ultrasonic LPS, GPS, *etc*.), since we, as well as other authors [20], believe that the definitive solution to the accurate and continuous indoor localization will require the integration of multiple technologies and algorithms in a hybrid location system.

## Figures and Tables

**Table 1.** Main features of the ramps in CAR-CSIC building.

**Ramp ID**	**Slope (°)**	**Size (m) (Length×Width)**	**Height (m)**	**Orientation**
1	11.3	2 × 4	0.4	North
2	3.8	3 × 3	0.2	East
3	3.2	3.5 × 2	0.2	West
4	2.8	2 × 1	0.1	East
5	4.1	5.5 × 3.6	0.4	West

**Table 2.** Analysis of probabilities for correct ramp detection, mis-detection and false alarm, as a function of the threshold (Th) used to detect a ramp.

**Th**	**Correct Detections**						**Mis-Detections**						**False Alarms**

	**R1**	**R2**	**R3**	**R4**	**R5**	**Total**	**R1**	**R2**	**R3**	**R4**	**R5**	**Total**	**Total**
5	100%	100%	100%	100%	100%	**100%**	0%	0%	0%	0%	0%	**0%**	**2.9%**
9	100%	100%	100%	76%	100%	**92%**	0%	0%	0%	24%	0%	**8%**	**0.15%**
15	100%	74%	100%	51%	100%	**85%**	0%	26%	0%	49%	0%	**15%**	**0%**
30	100%	49%	64%	0%	100%	**63%**	0%	51%	36%	100%	0%	**37%**	**0%**
